# Speckle-based high-resolution multimodal soft sensing

**DOI:** 10.1038/s41598-022-17026-0

**Published:** 2022-07-30

**Authors:** Sho Shimadera, Kei Kitagawa, Koyo Sagehashi, Yoji Miyajima, Tomoaki Niiyama, Satoshi Sunada

**Affiliations:** 1 Graduate School of Natural Science and Technology, Kanazawa University, Kakuma-machi, Kanazawa, Ishikawa 920-1192 Japan; 2 College of Science and Engineering, Kanazawa University, Kakuma-machi, Kanazawa, Ishikawa 920-1192 Japan; 3 Faculty of Mechanical Engineering, Institute of Science and Engineering, Kanazawa University, Kakuma-machi, Kanazawa, Ishikawa 920-1192 Japan; 4 Japan Science and Technology Agency (JST), PRESTO, 4-1-8 Honcho, Kawaguchi, Saitama 332-0012 Japan

**Keywords:** Optical sensors, Imaging and sensing

## Abstract

Skin-like soft sensors are key components for human–machine interfaces; however, the simultaneous sensing of several types of stimuli remains challenging because large-scale sensor integration is required with numerous wire connections. We propose an optical high-resolution multimodal sensing approach, which does not require integrating multiple sensors. This approach is based on the combination of an optical scattering phenomenon, which can encode the information of various stimuli as a speckle pattern, and a decoding technique using deep learning. We demonstrate the simultaneous sensing of three different physical quantities—contact force, contact location, and temperature—with a single soft material. Another unique capability of the proposed approach is spatially continuous sensing with an ultrahigh resolution of few tens of micrometers, in contrast to previous multimodal sensing approaches. Furthermore, a haptic soft device is presented for a human–machine interface. Our approach encourages the development of high-performance smart skin-like sensors.

## Introduction

Skin is the largest organ in the human body and enables humans to sense various physical stimuli to obtain information regarding their surrounding environment. Advanced technologies to build electrical or optical components on soft and stretchable substrates, i.e., flexible electronics^1–8^ and flexible photonics^9–20^, have been developed to mimic or extend the capabilities of biological skin. These technologies have led to new opportunities for numerous technical applications, including wearable electronics^7,21–23^, augmented reality^24^, prosthetic skins^25^, and soft robotics^26^. In the past decades, significant progress has been made to develop sensing capabilities with high sensitivity, high resolution, and fast response in flexible and stretchable substrates with a large area^27^. Different sensing mechanisms have been proposed and demonstrated, including electrical signal transduction strategies such as resistive^8^, capacitive^1^, piezoelectric^2^, and triboelectric^3^ methods, or detection strategies based on optical waveguide structures, such as stretchable waveguides^16^, optical fibers^17–19^, and fiber Bragg gratings^28,29^. Although these sensing mechanisms can play a crucial role in detecting different types of physical stimuli, most of the currently available sensors focus on detecting only a single physical stimulus and cannot distinguish between multiple stimuli simultaneously. Multimodal sensing capability, i.e., simultaneous sensing of different types of stimuli applied to sensors, is important for robots to efficiently perceive the physical world. Recently, considerable effort has been devoted to develop multimodal sensing or multi-functional sensing^2,20,27,30–35^. Such cases generally require multiple sensing elements with different sensing mechanisms to be integrated in a single sensing platform, which may need complex fabrication processes and/or may experience interference from other stimuli. Furthermore, to attain high-level perception such as object recognition, the spatial distribution of physical stimuli over a large-scale area should be measured with high spatial resolution. A previous approach to achieve high spatial sensing capability was based on the integration of a large number of sensors to form a sensor matrix with numerous wire connections; however, this usually incurs high integration complexity. Although a vision-based tactile sensing approach using a marker displacement is easy to manufacture^36^, the spatial resolution is limited, and it is difficult to simultaneously detect other parameters such as temperature.

Here, we propose a sensor-integration-free, flexible, high-resolution, and multimodal sensing approach, which does not require users to package or integrate different sensing materials; instead, the users can utilize a soft material as a multimodal sensing element without the formation of sensor arrays or sensor matrices and with minimal wiring. Consequently, this approach is potentially capable of large-scale spatial multimodal sensing. The unique feature of the proposed approach is based on the use of the optical scattering caused in a soft material, which is highly sensitive to external stimuli and allows the encoding of different stimuli as a spatial interference pattern. By using a data-driven decoding technique, users can freely decode various stimulus signals simultaneously. We demonstrate the simultaneous sensing mode of different physical quantities and the recognition mode for the shape of the contacting object. We also present a haptic soft-interface device. Our sensing approach reveals a novel pathway for high-performance skin-like sensors.

## Results

### Optical multimodal sensing methodology

**Figure 1:**
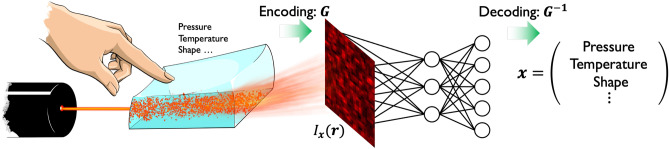
Conceptual schematic of the proposed soft sensing approach. The optical scattering phenomenon inside a soft material can induce a complex interference pattern, i.e., speckle pattern, which is highly sensitive to external stimuli on the material. The information on external stimuli, ${{{\textbf {x}}}}$, can be encoded as the speckle pattern, $I_{{{{\textbf {x}}}}}({{{\textbf {r}}}})$. The proposed sensing approach is based on the speckle encoding and decoding using machine learning.

The proposed sensing approach is based on optical scattering from soft materials when the material is irradiated with laser light (Fig. 1) The optical scattering produces a complex interference pattern, referred to as a speckle pattern^37,38^, on an observation plane. The speckle pattern is highly sensitive to the scattering process in a material; thus, it can contain various information regarding the deformation of a soft material. Although speckle-based techniques have been used in sensing a single physical signal^39,40^, in deformation field measurements^41,42^, three-dimensional shape measurements^43,44^, and in spectrometers^45^, this study presents the first demonstration of multimodal sensing in soft materials.

Here, we introduce a vector representing the physical parameters of the external stimuli to the soft material as ${{{\textbf {x}}}}= (x_1, x_2, \ldots , x_M) \in {{\mathbb {R}}}^M$. The speckle intensity pattern measured at the position ${{{\textbf {r}}}}$ on an observation plane is denoted as $I_{{{{\textbf {x}}}}}({{{\textbf {r}}}})$. Note that the stimulus information is optically encoded in a high-dimensional feature space as a spatial pattern, ${\varvec{G}}: {{{\textbf {x}}}}\rightarrow I_{{{{\textbf {x}}}}}({{{\textbf {r}}}})$. The spatial pattern can be regarded as the *optical neural response* to external stimuli. ${{{\textbf {x}}}}$ can be decoded from the neural response pattern $I_{{{{\textbf {x}}}}}({{{\textbf {r}}}})$ by identifying an inverse function, represented by ${\varvec{G}}^{-1}: I_{{{\textbf {x}}}}({{{\textbf {r}}}}) \rightarrow {{{\textbf {x}}}}$. This can be achieved using a learning-based model. Therefore, our sensing approach is a model-free (data-driven) approach, which does not require detailed theoretical models of the soft material and optical scattering. In addition, note that this approach does not require the integration of different types of sensors to detect multiple parameters, ${{{\textbf {x}}}}$; a single soft material acts as a sensing unit to separately estimate the multimodal stimulus information simultaneously.

### Proof-of-concept experiment

**Figure 2:**
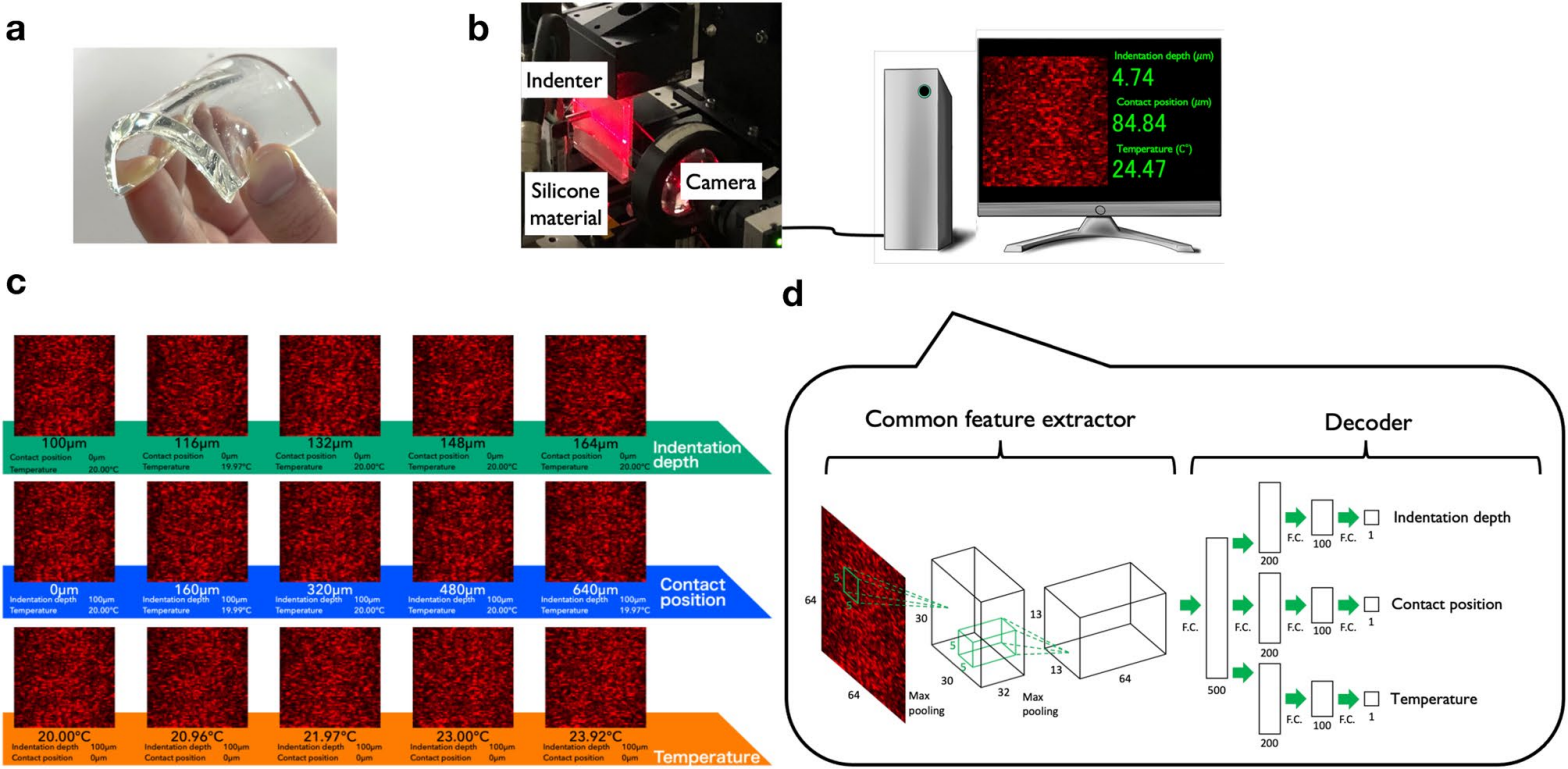
Experiment of speckle encoding and decoding. **(a)** Silicone material used in the experiment. **(b)** Experimental setup. The speckle pattern is measured with a digital camera and processed with the network architecture shown in (d). The decoded information ${{{\textbf {x}}}}$ (indentation depth, contact position, and temperature in this case) are displayed in real time with a monitor. **(c)** Speckle patterns produced by optical scattering from the silicone material. The patterns vary depending on the deformation of the silicone material and temperature. **(d)** Proposed deep learning architecture. It comprises the common feature extractor and decoder (regression section).

We performed an experiment for verifying the proposed sensing approach (See “Methods” section for details). The sensing targets of the experiment were tactile and thermal sensations. We chose a commercial transparent silicone elastomer material as the sensing material (Fig. 2a). The laser light was incident on the silicone material and scattered by impurities or less-visible air bubbles inside the material. The scattered intensity distribution at ${{{\textbf {r}}}}$ on an observation plane $I_{{{{\textbf {x}}}}}({{{\textbf {r}}}})$ was measured using a digital camera (Fig. 2b). In this experiment, a stainless cylindrical indenter was used to apply a normal force to the silicone elastomer. The position of the indenter was controlled with a positioning stage, and the indentation depth and contact location of the indenter were measured. The indentation depth was related to the force applied to the silicone material (Supplementary Fig. S1); therefore, we used the indentation depth as a substitute for the applied force. The measured speckle pattern $I_{{{{\textbf {x}}}}}({{{\textbf {r}}}})$ were sensitive to the indentation depth, location of the indenter, and temperature, as shown in Fig. 2c. The temperature-dependence of the speckle pattern can be attributed to thermal expansion/contraction or change in the refractive index. The features representing the physical stimuli, which are embedded in the speckle patterns, can be visualized using a nonlinear dimensionality reduction technique^46^ (Supplementary Fig. S2).

### Network architecture

Figure 2d shows the neural network model used to infer ${\varvec{G}}^{-1}$ and to simultaneously estimate ${{{\textbf {x}}}}= (x_1,x_2,x_3)$, where $x_1$, $x_2$, and $x_3$ correspond to the indentation depth, position of contact along a line in a sensor coordinate, and temperature, respectively. The network model comprises two components: (i) a common feature extractor, which extracts relevant common features from the speckle images and (ii) a decoder (regression model) to transform the extracted feature into physical quantities, ${{{\textbf {x}}}}$. The common feature extractor mainly consists of two convolution neural network (CNN) layers and a single fully connected (FC) layer, whereas the decoder consists of branched FC layers for the transformation into each physical quantity. See “Methods” section for the detailed structure.

### Multimodal sensing

**Figure 3:**
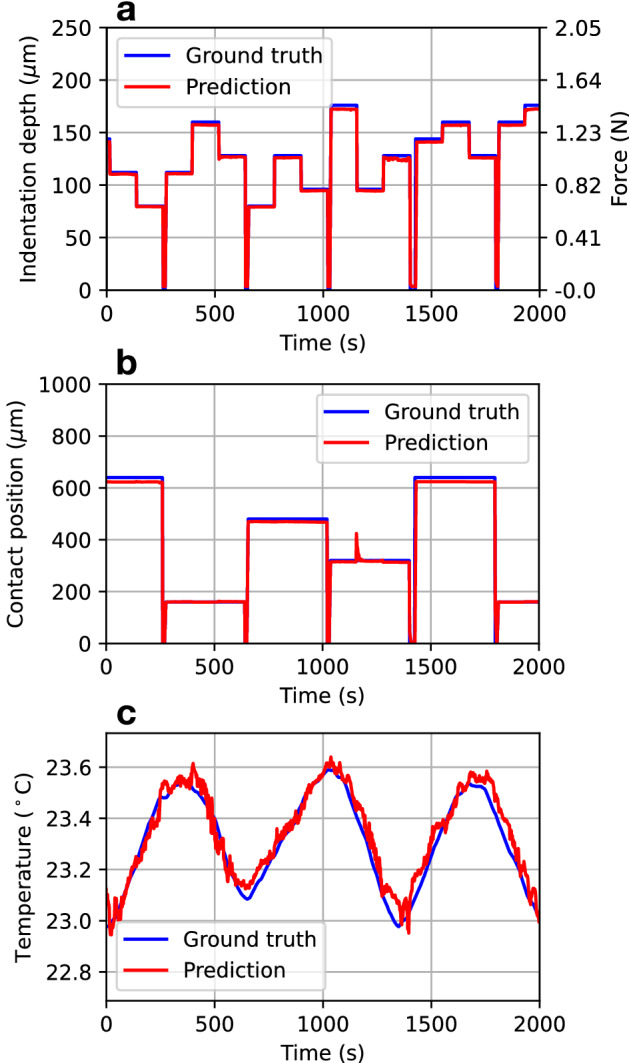
Multimodal sensing demonstration. Simultaneous estimations of **(a)** indentation depth, **(b)** contact position, and **(c)** temperature of the silicone material. In **(a)**, the applied force corresponding to the indentation depth is also shown. In **(b)**, an instantaneous large error at 1156 s is mainly attributed to an unintentional deviation from the set position of the indenter. The sensing data points were sampled at time steps of approximately 1.6 s.

**Figure 4:**
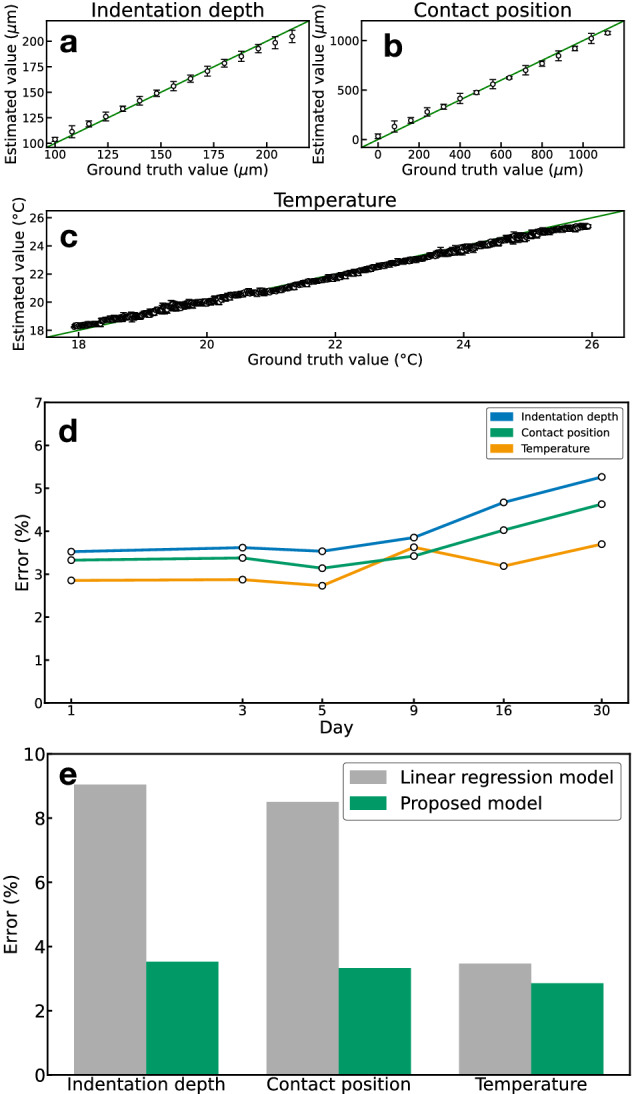
Sensing performance. With our sensing approach, we simultaneously estimated the indentation depth, contact position, and temperature of the silicone material. The estimated values of **(a)** the indentation depth, **(b)** contact position, and **(c)** temperature were compared with ground truth values. The error bars represent standard deviations. **(d)** Long-term stability of our sensing approach. The increase in the estimation errors was within 1.7 $\%$ for 30 days after the training. **(e)** Model comparison. The estimation errors of the proposed model shown in Fig. 2d are compared with a linear regression model, which does not contain hidden layers.

The network model was trained with *N* training data samples $\{I_{{{{\textbf {x}}}}}^{(n)}({{{\textbf {r}}}}),{{{\textbf {x}}}}^{(n)}\}_{n=1}^N$ (see “Methods” section), and used for multimodal sensing of the indentation depth, contact position, and temperature. Figure 3 illustrates the proposed multimodal sensing, where the network model was trained with $N =4000$ training samples. (See also Supplementary Video 1 for a real time demonstration of the multimodal sensing.) The simultaneous estimations of $x_1$, $x_2$, and $x_3$ could be achieved with a latency of only a few hundred milliseconds (Supplementary Fig. S3) with the performance of our computer (Supplementary Table S1), even when the depth and location of the indentation change in a random manner and the surrounding temperature varies under the effect of an air conditioner. Then, the network model was further trained with $N = 15,360$ training samples for better estimation. The performance of the simultaneous estimation was evaluated with 18, 000 test cases, where the indentation depth $x_1$ and contact position $x_2$ were changed at intervals of 8 µm and 80 µm, respectively. The temperature $x_3$ was controlled using a Peltier device. The estimation results are summarized in Fig. 4a–c. The estimation errors for the indentation depth, position, and temperature were ±3.95 µm (corresponding to ±32 mN, Supplementary Fig. S1), ±37.25 µm, and ±0.23 °C, respectively. The relative errors, defined as $\langle |x_{i}^{(n)} - {\hat{x}}_{i}^{(n)}| \rangle /(x_{i,max}-x_{i,min}) \times 100$ ($i =1, 2, 3$), were estimated as 3.52 $\%$, 3.33 $\%$, and 2.85 $\%$, respectively, where ${\hat{x}}_i^{(n)}$ is the estimated value for $x_i^{(n)}$ of the *n*-th sample, and $\langle \cdot \rangle$ denotes the sample mean. The errors were close to the precision of positioning of the indenter and temperature controls used in this experiment.

To evaluate the long-term stability of the proposed sensing approach, we recorded the time transition of the estimation errors for 30 days, as shown in Fig. 4d. In this experiment, we used the training dataset acquired on the first day and set the network parameters of the model shown in Fig. 2d; then, we measured the estimation errors. Although the speckle pattern measurement is generally sensitive to environmental changes, particularly temperature change, our results reveal that the error increased only by 1.7 $\%$ over 30 days and did not significantly change for 5 days, suggesting the robustness of our measurement method. This is attributed to the fact that the network architecture is trained for temperature changes.

To investigate the performance of the proposed deep learning model shown in Fig. 2d, we compared the model with a simple linear regression model, which does not contain hidden layers. In the linear regression model, the output vector ${{{\textbf {x}}}}$ is produced directly from the weighted summation of the input speckle images. As shown in Fig. 4e, the estimation errors of the proposed model are smaller than those of the regression model, suggesting the effectiveness of the CNN-based common feature extraction from the hidden layers in the proposed model. Thus, the model is superior to the linear regression model.

**Figure 5:**
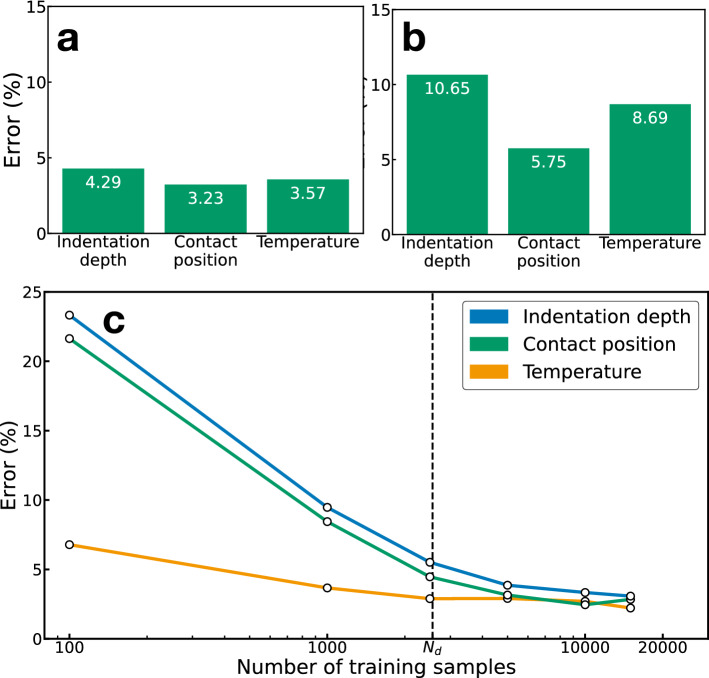
Effects of training data collection. Estimation errors for **(a)** $C(\Delta x_1) \approx 0.61$ and $C(\Delta x_2) \approx 0.66$ and **(b)** $C(\Delta x_1) \approx 0.42$ and $C(\Delta x_2) \approx 0.36$. The estimation errors depend on the sampling intervals $\Delta x_i$ (and the correlation $C(\Delta x_i)$ between the sampled speckle patterns). The estimation errors tend to be lower for smaller sampling intervals and higher correlations. **(c)** Estimation errors as a function of the number of training samples.

Training data collection, i.e., the method for collecting the training data samples, is crucial for precise estimations using the proposed sensing approach. Considering that the regression (interpolative estimation) is based on the training using correlated samples, the sampling interval, $\Delta x_i$, for the training data of $x_i$ ($i=1, 2, 3$) should be tuned such that the speckle patterns are correlated between the training samples. If the sampling interval is too large for the training samples to be uncorrelated, the interpolative estimation between the sampling intervals will be generally difficult. To investigate the effect of the sampling interval on the estimation performance, we changed the sampling intervals, $\Delta x_1$ and $\Delta x_2$, for the indentation depth and contact position and characterized the speckle correlation as $C(\Delta x_i) = \langle (I_{x_i}-{\bar{I}}_{x_i})(I_{x_i+\Delta x_i}-{\bar{I}}_{x_i+\Delta x_i})\rangle /(\sigma _{x_i}\sigma _{x_i+\Delta x_i})$ ($i=1, 2$), where ${\bar{I}}_x$ and $\sigma _x$ are the mean and standard deviation of the speckle intensity pattern, respectively. As shown in Fig. 5, the estimation errors depend on the speckle correlation $C(\Delta x_i)$. For $\Delta x_1$ = 12 µm and $\Delta x_2$ = 120 µm, the speckle correlations were $C(\Delta x_1) \approx 0.61$ and $C(\Delta x_2)$ $\approx 0.66$, respectively. In this case, the estimation errors were 4.29 $\%$, 3.23 $\%$, and 3.57 $\%$ (Fig. 5a). When the sampling intervals were large ($\Delta x_1$ = 52 µm and $\Delta x_2$ = 520 µm), $C(\Delta x_1)$ and $C(\Delta x_2)$ decreased to 0.42 and 0.36, respectively, and the mean estimation error increased to approximately 10.65 $\%$, 5.75 $\%$, and 8.69 $\%$ (Fig. 5b), which suggests that the speckle correlation between the samples affects the generalization capability of the proposed model.

It is also important to consider the appropriate training data size for good estimation performance with low training cost. Figure 5c shows the estimation errors as a function of the number of training samples, *N*. As seen in this figure, the estimation errors sufficiently decrease when $N > N_d = 2560$, as indicated by the vertical dotted line, where $N_d$ is the number of training samples that cover all states of the soft material in response to indentation and temperature changes in this experiment (see “Methods” section). The oversampling for $N > N_d$ can further lower the errors, suggesting that the errors caused by laser noise and temperature fluctuation can be compensated.

### Sensing and perception

**Figure 6:**
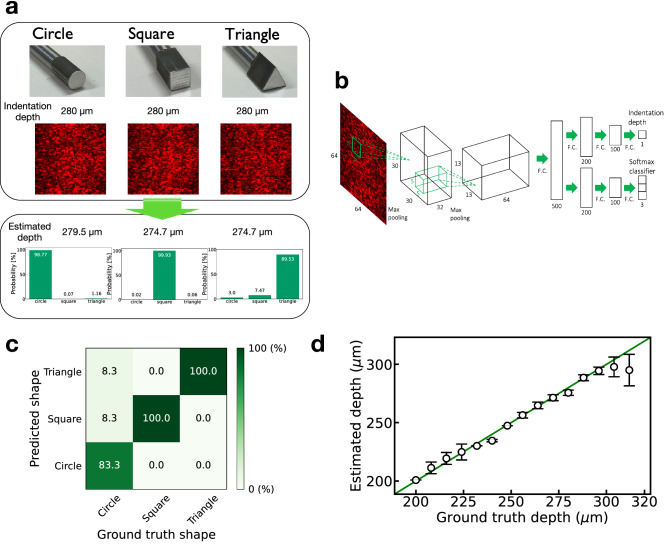
Simultaneous realization of sensing and perception. **(a)** Three indenters with circular, square, and triangular cross sections were used in the experiment. The speckle patterns change depending on the shapes of the indenters and contact force on the soft material. Examples of the speckle patterns for each indenter with an indentation depth of 280 µm are shown in **(a)**. The indenter shape and depth can be identified in the network model shown in **(b)**, where the common feature extractor is the same as that in the model shown in Fig. 2d. The network can be trained to reduce both the mean squared error and cross entropy with 405 training samples. The estimation results for 45 test samples are shown in **(c)** and **(d)**. **(c)** Confusion matrix of shape identification. **(d)** Estimation of the indentation depth.

As mentioned above, the speckle patterns are highly sensitive to various external stimuli on the soft material, that is, they contain the information on various external stimuli. This suggests that the information on multiple external stimuli, which is difficult to detect using a single-mode sensor (e.g., pressure sensor or temperature sensor), can be extracted from the speckle patterns. In addition, this results in the advantage that it is not necessary to rebuild the sensing device when changing the target stimuli; instead, it is sufficient to modify the training and post-processing of the sensing in our sensing approach. As a demonstration, we selected various shapes of the indenters used to deform the soft material as the sensing targets and verified the identification of the shapes of the indenters along with the sensing of the indentation depth (corresponding to the contact force). In the experiment, we used three types of indenters with circular, square, and triangular cross-sections (Fig. 6a); the areas of the cross-sections were equal to each other. Although shape identification generally requires spatial information of the deformation, which cannot be detected using a single-point measurement, our sensing approach can optically grasp the spatial information with a high spatial resolution of few tens of micrometers (Fig. 4). The shape identification can be easily achieved using a shape classifier in the decoder section of the network architecture, as shown in Fig. 6b. The network model can be trained such that the mean squared error for the regression of the indentation depth and cross entropy for the classification of the indenter shape are both minimized. Figure 6c,d show the simultaneous estimation results of the indenter shapes and indentation depths (also, the indentation force). The experiment used 450 samples (90$\%$ and 10$\%$ of the total samples were used for training and testing, respectively). The identification error was approximately 5.6$\%$, and the mean estimation error of the indentation depth was approximately 3.8$\%$ (corresponding to a precision of 4.2 µm). The network model is highly scalable for the number of estimated physical quantities; thus, using a larger number of training samples can enable simultaneous estimation of additional physical quantities with lower errors.

### Toward human–machine interface

**Figure 7:**
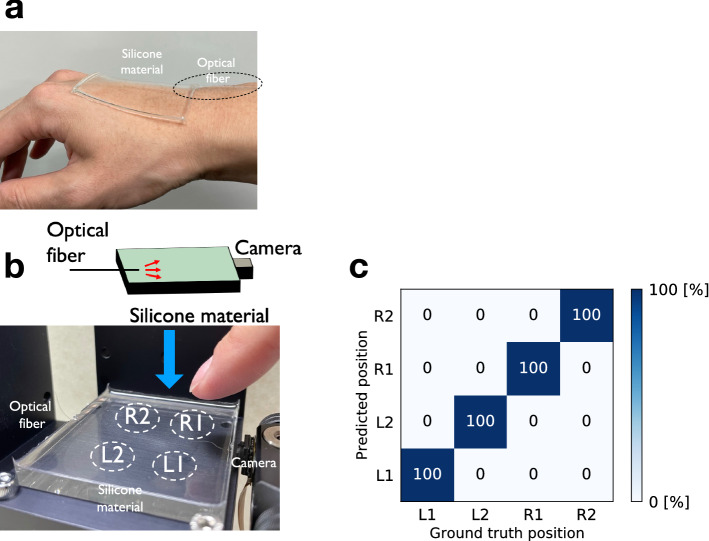
Human–machine interface. The interface device consists of a thin transparent soft material, optical fiber, and miniature camera. **(a)** A thin silicone material can be fitted on the human hand. **(b)** Interface device. The device can detect four positions, indicated by *L*1, *R*1, *L*2, and *R*2, where the silicone material is pressed by a human finger. **(c)** Classification accuracy. The contact position can be identified with 100 $\%$ accuracy after training.

For application in a human–machine interface, the proposed optical sensing unit can be easily incorporated with an optical fiber to deliver the laser light to the soft material along with a miniature camera for detection. A thin skin-like silicone material can be fitted onto the human body to allow physical sensing and controlling (Fig. 7a). More importantly, the proposed optical sensing approach allows us to control the sensitivity for detecting the external stimuli via speckle patterns. We attempted to change the sensing precision from the micrometer scale, demonstrated above, to the millimeter scale, which is more suitable for detecting touching motions. Such a sensitivity reduction can be achieved mainly by detecting single scattering or low-order multiple scattering light signals, which are less sensitive to deformation, with the reduction of the laser power and camera sensitivity, considering that high-order scattering light is generally weaker than low-order scattering light. Figure 7b shows our proposed soft interface device, which consists of a transparent silicone material, optical fiber, and miniature digital camera. The laser power and exposure time of the camera were tuned such that higher-order scattering signals were less detectable; thus, only low-order scattering signals could be detected. The silicone material was pressed by a human finger at four positions, indicated by *L*1, *L*2, *R*1, and *R*2, and the speckle patterns were measured using a miniature camera. The contact positions were successfully identified after the training using 320 samples (Fig. 7c). The complete identification is partly due to the simple task of classifying the stimuli into a few classes instead of considering the multimodal regression problem.

## Discussion

In this work, we demonstrated an optical multimodal sensing approach, which enables highly sensitive simultaneous sensing of physical contact and temperature changes, with the additional advantages of low electromagnetic interference, noninvasive nature, high stability, and low-cost design. The multimodality is a remarkable feature of our sensing technique, in contrast to single-modal sensing techniques, which provide no information on the other physical quantities. The high sensitivity originates from the optical scattering interference, which is based on the responses of the speckle patterns to physical stimuli. The sensing mechanism is entirely different from previous optical sensing approaches, which used the changes in the light intensity and wavelength. It should be emphasized that our sensing approach can be applied to various optical materials because the only prerequisite is the creation of a speckle phenomenon in the sensing material. Owing to the high sensitivity realized with the optical scattering interference, the proposed approach can enable the sensing of physical contacts with high spatial resolution of few tens of micrometers. Thus, it outperforms previous electrical and optical approaches used in this field of study^33–36^, although the precision is slightly lower than that of the state-of-the-art measurements, such as speckle interferometry, holography, and projection techniques^42,44,47,48^. Higher spatial resolution and greater precision of measurement of the indentation can be achieved by using a larger number of training samples. An opaque material, which induces higher-order multiple scattering, can facilitate speckle-based sensing with greater sensitivity.

Regarding the implementation of the multimodal sensing principle, we emphasize that the proposed approach is superior to previous multimodal sensing approaches, as it avoids complex integration of multiple sensing elements with different configurations or designs of sensor networks (sensor matrices) into a single sensing device with numerous wire connections. This unique property would be useful in seamlessly integrating the skin-like sensors with soft actuators for realizing soft robotics.

Despite the advantages of the proposed approach, there is room for further improvement. The main drawback is the requirement of a large-scale model with a large number of training samples for multimodal sensing, which may make real-time operation difficult when multiple parameters need to be estimated. One way of overcoming this drawback is to prepare an ensemble of models that are separately trained for different datasets and to distill the knowledge to be transferred from the trained model to a small model. This may be possible by adopting a transfer learning technique or knowledge distillation^49^. Knowledge distillation also enables the small model to be trained on much less data^49^.

Because of the high information capacity of the speckle patterns, the proposed approach can enable higher-level perception and higher multimodality, including the multimodal sensing of shear force, distortion, and three-dimensional shape of a contact object, which are difficult to sense using conventional sensors but are important when considering robot hands to grip soft and slippery objects. Our model-free (data-driven) sensing strategy allows us to freely choose the sensing modes according to the purpose. An interesting topic for future work will be to realize soft sensing with nanometer-scale spatial resolution. Another interesting future work is to create sensory signals for fuzzy sensations such as touch, warmth, or pain for use in prosthetic sensory skin by integrating and processing multimodal sensory signals.

## Methods

### Experimental setup

A schematic of the proof-of-concept experiment is shown in Supplementary Fig. S4. We used a commercial transparent soft silicone material (Verde Co., Ltd., Superclear silicone). The physical dimensions of the silicone material were 58 mm$\times$52 mm$\times$5 mm. A He-Ne laser (MELLES GRIOT, 05-LHP-991, wavelength 632.8 nm, beam diameter 0.65 mm) was used as the light source. The speckle size of the measured speckle pattern can be controlled by varying the distance from the scattering location to the observation plane, illumination area (beam diameter), and the pupil diameter of the imaging lens. In the experiment, the distance was set to approximately 10 cm. The lens diameter and focal length were 50 mm and 200 mm, respectively. The scattered intensity distribution was detected with a digital camera (Thorlabs, DCC1240C) with exposure time of 5 ms. The silicone material was deformed using a stainless cylindrical indenter with contact area diameter of 3 mm. The depth $x_1$ and location $x_2$ of the contact with the indenter were automatically controlled using a two-axis stepping motor controller with a precision of $\pm 0.5$ µm. We determined the origin of the indentation depth by moving the indenter until the contact could be observed. The contact position $x_2$ was moved along a vertical line. The temperature $x_3$ of the silicone material was controlled using a Peltier device with a thermistor embedded inside the silicone material for temperature monitoring (Supplementary Fig. S4). (The precision of temperature control was estimated as ±0.2 °C.)

The sensing unit shown in Fig. 7b consists of a transparent silicone material, polarization-maintaining optical fiber (Thorlabs, PM630-HP), and miniature digital camera with angle view of 62$\times$47 degrees (ArduCam, OV5647 Spy Camera Module for Raspberry Pi). The total input power of the laser light was less than 1 mW, and the exposure time of the camera was set to 1/8 s. The length of the silicone material was limited by the power loss, which was mainly induced by absorption and scattering.

### Preprocessing and network model

For the preprocessing of the measured speckle images to be used as input images to the network model shown in Fig. 2d, the images were downsampled to 30 $\%$ and trimmed to 64$\times$64 pixel images. The kernel size of the downsampling was set to be close to the mean speckle size to reduce the sensitivity of the speckle patterns to environmental fluctuations such as vibration and air fluctuation. The resizing effects on the estimation errors are shown in Supplementary Fig. S5. Regarding the physical parameter vector ${{{\textbf {x}}}}= (x_1,x_2,x_3)$, each of the features, $x_1$, $x_2$, and $x_3$, was scaled to a range between − 1 and 1 in the training dataset, i.e., $x_i \rightarrow x_{s,i} = 2(x_i-x_{i,min})/(x_{i,max}-x_{i,min}) - 1$, $(i = \{1,2,3\})$, where $x_{i,max}$ and $x_{i,min}$ are the maximum and minimum values of $x_i$, respectively, to eliminate the difficulties that could result from different physical dimensions. Let ${{{\textbf {x}}}}_s = (x_{s,1},x_{s,2},x_{s,3})$ be the scaled vector, and by using a training dataset of *N* cases, $\{I^{(n)}_{{{{\textbf {x}}}}}({{{\textbf {r}}}}),{{{\textbf {x}}}}_s^{(n)}\}_{n=1}^N$, the network parameters were trained to minimize the following mean square error (MSE): MSE $= 1/N\sum _{n=1}^N({{{\textbf {x}}}}_s^{(n)}-{\hat{{{{\textbf {x}}}}}}_s^{(n)})^2$, where ${\hat{{{{\textbf {x}}}}}}_s^{(n)}$ is the output vector for the *n*th sample. The inferred parameter vector ${\hat{{{{\textbf {x}}}}}}^{(n)}$ can be obtained from the inverse transformation of the min-max scaling, ${\hat{{{{\textbf {x}}}}}}_s^{(n)} \rightarrow {\hat{{{{\textbf {x}}}}}}^{(n)}$. The mini-batch size for the gradient-based optimization was set to 50, and the Adam optimizer was used.

In the deep learning model shown in Fig. 2d, the first CNN layer uses 32 kernels of size $5\times 5$ and the ReLU activation function, followed by batch normalization and max pooling of size $2 \times 2$ and stride 2. The second CNN layer uses 64 kernels of size $5 \times 5$ and the ReLU activation function, followed by batch normalization and max pooling of size $2 \times 2$ and stride 2. The features are shared in three branched networks, which consist of three FC layers. FC matrices of size $500 \times 200$, $200 \times 100$, and $100\times 1$ were used in the first, second, and third FC layers, respectively.

In the deep learning model shown in Fig. 6b, the common feature extractor is the same as in the model shown in Fig. 2d. In the decoder section, a classifier was added for shape identification. The network was trained such that both the MSE for the regression and cross entropy for the classification were minimized.

### Data collection protocol

To collect the data to train the network model shown in Fig. 2d, we used a two-axis stepping motor controller and changed the indentation depth from $x_1 =$100–212 µm with a sampling interval of $\Delta x_1 =$ 16 µm, and the contact position from $x_2 =$ 0–1120 µm with an interval of $\Delta x_2 =$ 160 µm, where the origin of $x_1$ (pressing depth of the indenter) was set as the surface of the silicone material. The temperature was changed from $x_3 =$17.9–26.0 °C with an interval of 0.2 °C using a Peltier device and/or air conditioner. We simultaneously recorded the speckle pattern $I_{{{{\textbf {x}}}}}({{{\textbf {r}}}})$ for each stimulus. The number of training samples obtained in this process, $N_d$, was $\sim$2560. We repeated this sampling process and set $N/N_d \approx 6$ to reduce the fluctuation in the measured speckle patterns and improve the robustness of the network to noise, where $N(\approx 6N_d) = 15,360$ is the total number of training samples used for the proof-of-concept experiment (Fig. 4).

## Supplementary Information

Supplementary Video 1.

Supplementary Information.

## Data Availability

The data that support the findings of this study are available from the corresponding author upon reasonable request.
