# Supplementary material for: Speckle-based high-resolution multimodal soft sensing
Source: Sci Rep. 2022 Jul 30;12:13096. doi: 10.1038/s41598-022-17026-0 (PMC9338967; doi:10.1038/s41598-022-17026-0)
Supplement: Supplementary file 2 — Supplementary Information. [file 41598_2022_17026_MOESM2_ESM.pdf]

Supplementary Information:
Speckle-based high-resolution multimodal soft sensing

Sho Shimadera[1], Kei Kitagawa[2], Koyo Sagehashi[1], Yoji Miyajima[3], Tomoaki Niiyama[3], and Satoshi Sunada[3,4*]

[1]*Graduate School of Natural Science and Technology, Kanazawa University, Kakuma-machi, Kanazawa, Ishikawa 920-1192, Japan*

[2]*College of Science and Engineering, Kanazawa University, Kakuma-machi, Kanazawa, Ishikawa 920-1192, Japan*

[3]*Faculty of Mechanical Engineering, Institute of Science and Engineering, Kanazawa University Kakuma-machi, Kanazawa, Ishikawa 920-1192, Japan*

[4]*Japan Science and Technology Agency (JST), PRESTO, 4-1-8 Honcho, Kawaguchi, Saitama 332-0012, Japan*

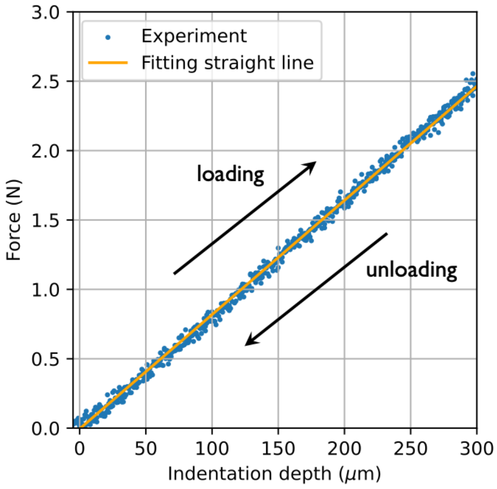

FIG. S1. **Mechanical properties of the silicone material.** We performed an indentation experiment for the silicone material in both loading and unloading regimes. In this test, a stainless cylindrical indenter with the contact area diameter of 3 mm was used. The dimension of the sample used in this experiment was same as that of the sample shown in Fig. 2 in the main text. The sample was pressed by the indenter at a step of 1 μm. The applied force was measured with a compression load cell (KYOWA, LUX-B-50N) and was shown as a function of indentation depth in the loading and unloading regimes. Note that the hysteresis between the loading and unloading regimes in the experimental result was not significant. We fitted the experimental data with a linear model. The slope of the best fitting line was 0.00823 N/μm. We estimated the applied forces corresponding to the measured indentation depth (shown in the main text) by using the best fitting line.

* sunada@se.kanazawa-u.ac.jp

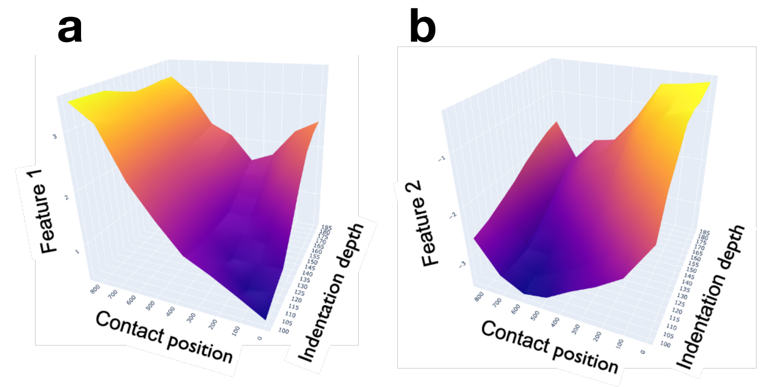

FIG. S2. **Two-dimensional projection of the speckle image dataset.** The speckle images were projected onto a two-dimensional feature space for visualization by a nonlinear dimensionality reduction technique (t-distributed stochastic neighbor embedding, t-SNE). The perplexity was set to 50. In **a.** and **b.**, two features 1 and 2 embedded in the speckle patterns are shown as functions of the indentation depth and contact position. The features continuously change depending on the physical stimuli, which suggests that they can be extracted from the speckle patterns using a decoder.

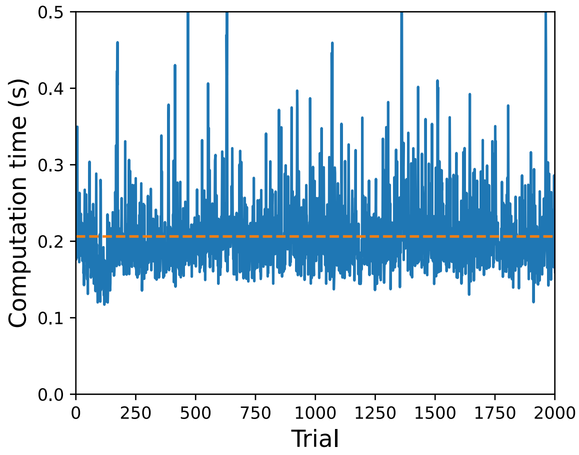

FIG. S3. **Computation time for 2000 trials.** We measured the total computation time, including the preprocessing of the captured speckle images and computation of the proposed neural network model. The specifications of the computer used in the experiment are summarized in Table S1. The mean computation time for 2000 trials was approximately 206 ± 50 ms. The dashed line denotes the mean computation time of 206 ms. Because the simultaneous estimations of three parameters can be achieved with a latency (time delay) of few hundred milliseconds [Fig. 2 in the main text], we speculate that the latency is mainly due to the computation time between the preprocessing of the captured speckle images and output of the proposed model.

TABLE S1. **Specifications of the computer used in the experiment.**

Item	Value
OS	Windows 10
CPU	Core(TM) i7-10700 CPU @ 2.90 GHz
RAM	16.0 GB
GPU	Geforce GTX 1650 SUPER

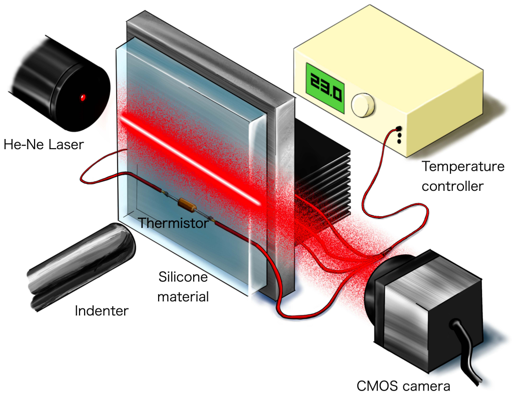

FIG. S4. **Schematic of the experimental setup for the results shown in Figs. 2-4 in the main text.** The silicone material is deformed using a stainless cylindrical indenter. The speckle pattern is measured with a CMOS camera. The thermistor is used to monitor the temperature. The recorded temperature is used for training the network model.

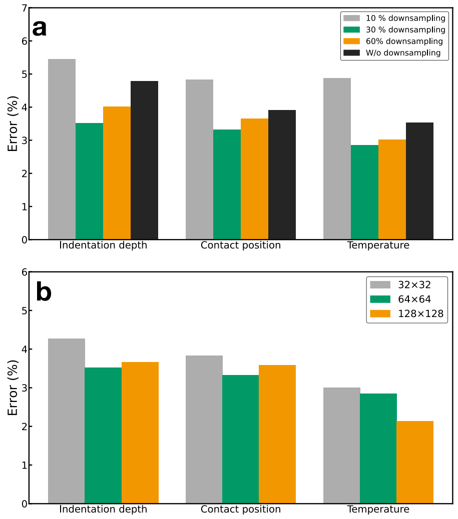

FIG. S5. **Resizing effects on estimation error.** The speckle images were downsampled and trimmed for the pre-processing. **a.** Effect of downsampling. The image size was fixed as 64×64-pixel size. The errors are minimized when the original images were downsampled to 30%. The large downsampling of 10% degrades the estimation performance. **b.** Effect of the image size on the estimation error. As the image size is reduced, the computation cost is reduced for the estimation but the performance is degraded. In this study, we used the speckle images of 64×64 pixel size.

Supplementary Video

Real-time demonstration of multimodal sensing. The right monitor displays the setting values, while the left monitor displays the estimated values.
